# Assessing food procurement greenhouse gas emissions and food waste in UK fine dining

**DOI:** 10.1016/j.clfs.2025.100010

**Published:** 2026-06

**Authors:** Andrea Zick, Ximena Schmidt R, Christian Reynolds

**Affiliations:** a Department of Chemical Engineering, Brunel University London, Uxbridge, UB8 3P, United Kingdom; b Centre for Food Policy, School of Health and Medical Sciences, City St George's, University of London, United Kingdom

**Keywords:** Supply chain management, Procurement management, Menu engineering, GHG emission reporting, Sustainable hospitality, Hospitality and food service (HaFS)

## Abstract

This research examines greenhouse gas emissions (GHGE) associated with food procurement and food waste in a UK fine dining restaurant. A comprehensive food procurement greenhouse gas emissions baseline was established, emission hotspots were identified, and food waste reduction targets previously set by the restaurant were verified.

A total of 6282 individual food purchases were reviewed. Due to repeat purchases, 941 distinct food and drink commodities were matched with 920 emissions factors from the WRAP Emission Factor Database v2.0, enabling a volume-based greenhouse gas emissions assessment. The analysis revealed seasonal variations and GHGE hotspots, providing a benchmark for similar catering operations. A Monte Carlo simulation was performed by randomising the emissions factors allocated to assess the sensitivity of the assessment. Despite the possible variation of emissions factors, the average GHGE per guest was found to be 5.87 kg CO_2_ eq. per guest (±0.27) and 4.79 kg CO_2_ eq. per kg of food procured (±0.54). A dietary gap analysis found the associated GHGE exceed the range for GHGE per day/person of the Eatwell guide recommended by the British Dietetic Association as a healthy, sustainable diet. The analysis also shows that GHGE associated with food waste represents the fourth-largest contributor.

Establishing a baseline for GHGE of food waste and procurement supports measurable goal setting, intervention identification, and progress tracking towards emission reduction targets. The findings equip the business to design targeted and evidence-based interventions.

## Highlights

• First GHGE baseline for UK fine dining food procurement and waste.
• Bovine meat, lamb, and fish drive 71 % of food-related GHGE.
• GHGE per guest exceeds Eatwell Guide targets year-round.
• Food waste is the fourth-largest GHGE contributor.
• Monte Carlo analysis shows 4 % GHGE underestimation.

## 1 Introduction

Increased environmental awareness has led to sustainability initiatives in the Hospitality and Food Service (HaFS) sector, focusing on food waste, energy, and water management (Robinson et al., 2024). Food production, transport, processing, consumption, and disposal can significantly impact greenhouse gas emissions (GHGE) in the food system (Willett et al., 2019). Forbes et al. (2021) stated that food and drink are responsible for 35 % of UK territorial GHGE. As an economically significant contributor to the UK food system (Hasnain et al., 2020), HaFS plays a role in shifting this environmental impact.

Key components for reducing GHGE in HaFS are ingredient selection, food offer changes, and food waste reduction. Global food waste in HaFS is estimated to be 244 Mt per year, with 19 % avoidable (United Nations Environment Programme, 2021). In the UK, food waste was estimated to be 10.7 Mt in 2021 (Malik et al., 2024), accounting for around 36 Mt of CO_2_ eq. (WRAP, 2021).

Business GHGE are assessed as scopes 1 and 2, and those from the procurement of goods and services are scope 3 emissions (BEIS, 2021). The contribution towards scope 3 varies between food-led hospitality businesses and those which focus on accommodation or events (Cerutti et al., 2018; Demeter et al., 2021; Dwyer et al., 2010; Filimonau et al., 2011; Mistretta et al., 2019). Various food-led HaFS businesses (e.g. Compass Group, 2023, Compass UK & Ireland, 2024; Sodexo, 2024; Azzurri Group Ltd, 2024; The Restaurant Group, 2021) publicly report on scope 3 emissions, which are a significant contributor to their total carbon footprint. Within scope 3 emissions in these businesses, food waste and procurement are major contributors. Other HaFS companies have yet to quantify their scope 3 emissions, and there have been no academic publications on small and medium enterprises, specifically fine dining restaurants. This study aims to fill this empirical gap by assessing all food-related procurement emissions and estimating the food waste emissions for a fine dining restaurant using the WRAP Scope 3 Measurement and Reporting protocols (WRAP, 2024). It also explores the feasibility of regularly reviewing GHGE using the WRAP methodology in smaller independent businesses and opportunities and barriers that may exist. The GHGE of food waste will be compared with the GHGE of the food procured.

Research suggests that people in temperate climates have different diet patterns throughout the year (Folwarczny et al., 2022; Spence, 2021). Folwarczny et al. (2022) argue that this is linked to environmental cues which suggest food scarcity, while Spence (2021) points towards cultural factors and marketing messages as the drivers of those seasonal diet pattern changes. Overall, studies suggest that energy intake and consumption of energy-rich foods increase towards winter and decrease near summer (Folwarczny et al., 2022). Thus, we hypothesise that these seasonal variations are evidenced in the GHGE of food procurement in a restaurant.

Finally, the procurement data provides an opportunity for a dietary gap analysis (Kelly et al., 2025), specifically in how far the proportions of food procured represent the proportions recommended by the Eatwell guidelines. To the authors' knowledge, such a dietary gap analysis in a fine dining SME in the UK has not yet been carried out, and thus it is a further novelty of this study.

## 2 Methodology

### 2.1 Conceptualisation

To allow comparison with the primary data from the GHGE analysis, an explorative grey literature search of scope 3 emission data from other food-led UK HaFS companies was carried out in June 2025 with the aim of creating a ‘baseline data set’ for small medium HaFS businesses. The author selected HaFS businesses randomly from the State of the Nation report 2024 (MacKean et al., 2024), which reviewed transparency on net-zero and scope 3 emission reporting in the UK of 36 food businesses (16 HaFS businesses). For the selected businesses, the respective impact reports were searched for online to extract relevant scope 3 emission data. While the report included five casual dining, five contract caterers and six Quick Service restaurants, there were no independent and small to medium HaFS businesses in this report, which would be more comparable with the business in question. After contacting the business's senior management, two firms were suggested, and it was recommended to explore the Michelin guide (Michelin, 2025) for similar businesses. The websites of the two enterprises suggested were searched for emissions disclosures; however, no relevant information was accessible. This was followed by a search of restaurants with a Green Michelin Star in the UK (n 5), and a search for vegetarian and plant-forward independent small restaurants' (n 10) emissions disclosure. The 15 websites of those businesses currently do not disclose their scope 3 emissions data; thus, this could not be included. In the absence of relevant competitor data, Azzurri Group Ltd (2024), Dishoom UK (2024) and Hawksmoor (2024) scope 3 emissions data were added to the scoping study as these businesses have similarities to the company that provided the procurement and food waste data and were not listed in the MacKean et al. (2024) report.

### 2.2 Data collection

In September 2024, 12 months of procurement (Jun 23-May 24), food waste (Sep 23-Aug 24), and visitor data from a sizeable fine-dining restaurant were accessed. The restaurant is based in a central city location, and typically only serves lunch and dinner. The restaurant is also available for event bookings, which occur several times per months, the average number of guests per day for the period observed was 444 (±70). The procurement data was retrieved from Tevalis (v2025.04.15.01). The food waste data was received in several Excel files, which used the Guardians of Grub Food Tracker (WRAP, 2017) and the visitor data was shared from Sevenrooms Inc. The restaurant had previously engaged Eco Veritas to estimate its scope 1 and 2 emissions and to conduct a sales-based scope 3 emission estimation; this assessment was also shared with the research team for context.

### 2.3 GHGE assessment

A screening inventory was created using WRAP's guidance for scope 3 emissions assessments (WRAP, 2024). The inventory referred to the scope 1, 2 reports, a sales-based scope 3 assessment received from Eco Veritas and engaged with the executive chef, procurement manager and the financial team to understand the data sources. This can be found in Appendix A.

Detailed steps for food procurement GHGE estimation are in Appendix B. The procurement data contained 6282 lines of purchases. The following steps were carried out: all lines of procured food recorded were converted to mass (kg). Purchasing data was cleaned (removal of lines with zero weight values n-60, removal of lines which were identified as human error n-34 and lines which did not link to a specific food commodity n-18). 941 repeat purchases (unique commodities) were identified. WRAP Emission Factor Database v2.0 (WRAP, 2024) was referenced for appropriate emissions factors for the UK. For some commodities (e.g. baking powder), no emissions factor was available; thus, 19 commodities amounting to 120 lines of data were removed. A total of 920 specific emissions factors were allocated because for some commodities, the same emissions factor was used, for example, for beef filet and beef shin purchases. The total annual GHGE were calculated, as well as monthly GHGE; monthly variations were statistically analysed with t-tests. The study includes two functional units: ‘the GHGE per guest’ and ‘the GHGE per kg of procured food’. The selection of these functional units is intended to provide restaurants with information for incorporating this indicator into internal processes that assess performance at guest level, while also enabling comparisons across other sectors.

To enable a hotspot analysis, each commodity was categorised into 40 GHGE groups. Previous research informed the choice of the GHGE categories, such as Clune et al. (2017), Jungbluth et al. (2016), as well as the GHGE reference database used for this study (WRAP, 2024). These studies and guidelines use different categories: Clune et al. (2017), 20 categories; Jungbluth et al. (2016), 10 categories; and WRAP (2024), 133 categories. To balance breadth and depth of the analysis, 40 categories were chosen in this study. The total annual GHGE for these 40 categories were calculated.

### 2.4 Monte Carlo sensitivity analysis

A Monte Carlo simulation for the procurement GHGE assessment was conducted, inspired by Reynolds et al. (2014) and Johanson and Evens (2007), where emissions factors from the WRAP Emission Factor Database v2.0 were simulated based on a set of coefficients of variation. The aim was to understand the extent to which the variation in emissions factors influences the results and to assess the sensitivity of the calculation. For the Monte Carlo simulation, random emission factors were generated for each of the 920 emission factors within a specified statistical distribution, using the following coefficients of variation: 0.21 for all pure animal proteins, 0.57 for all pure plant commodities, and 0.5 for mixed commodities (such as baked goods, e.g., almond croissants). The review by Clune et al. (2017) informed the choice of these coefficients of variation. The process of randomly allocating emissions factors within the set coefficients of variation and recalculating GHGE was repeated 4000 times to ensure robust results. A dataset was compiled containing the assigned GHGE per commodity per year and the total annual GHGE for each simulation.

Nine procurement categories from the raw business data set, which had been retained in the data which contained the GHGE emissions factor allocations, were used for the data analysis of the Monte Carlo simulation rather than 40 GHGE categories. The reason for that approach lies in the limited computational power. The Monte Carlo simulation had been carried out with MS Excel, and the processing memory of the system was at capacity. It was therefore more suitable to reduce the number of categories to compute.

### 2.5 Dietary gap analysis

Studies suggest that diets which are more closely aligned with national dietary guidelines (Kelly et al., 2025; Scheelbeek et al., 2020; Trewern, 2024) would support sustainable food systems transition; thus, comparing the procurement composition with the Eatwell guidelines could further help to identify opportunities for menu transformation. Kelly et al. (2025) used the percentage Eatwell guide contribution to review the compliance of the UK food supply. This study applies a similar approach using the annual food procurement data from the restaurant to assess how far the procurement composition matches the Eatwell guidelines. In addition, the estimated GHGE range for the Eatwell guideline (British Dietetic Association, 2020) is used throughout as a baseline indicator of achieving GHGE emission reduction targets.

After grouping commodities into GHGE 40 categories, those categories were allocated into six Eatwell guideline categories (starchy foods, fruit and vegetables, dairy and alternatives, protein, oils and spreads and ‘other’). Their proportionate procurement volume contribution was then calculated to enable percentage comparison with the Eatwell guide recommendations for a dietary gap analysis.

### 2.6 Food waste assessment

The restaurant staff recorded food waste data using the Guardians of Grub Food Tracker (WRAP, 2013) for 52 weeks (Sep 23 – Aug 24). Records were consolidated into thirteen four-week periods, calculating food waste and associated GHGE per guest. To make the procurement data and food waste data comparable, the GHGE for Sep 23 – May 24 for procurement categories and food waste per guest were calculated (totalling 36 weeks of comparable data). The Guardians of Grub emissions factor was used for food waste calculations (WRAP, 2017) due to the absence of a specific factor in the WRAP Emission Factor Database v2.0 (2024). The sum of 36 weeks of food waste per guest GHGE emissions was compared with the 40 GHGE category contributions for Sep 23 – May 24. Further detailed information can be found in Appendix C.

## 3 Results

### 3.1 Sector scope 3 emissions data

Table 1 summarises the findings of the explorative review of scope 3 emissions disclosure of UK catering businesses, confirming an empirical gap for UK SME catering businesses.

**Table 1:** Comparison of scope 3 emissions of catering businesses operating in the UK.

Company name^a^ (Year of report)	Yum! Brands, (2023)	Compass Group UK & Ireland (2024)	Sodexo (2024)	The Restaurant Group UK (2021)	Azzurri Group Ltd (2024)	Dishoom UK (2024)	Hawksmoor (2024)
**Type of business**	Quick Service Restaurants	Contract Caterers and Food Service	Contract Caterers and Food Service	Casual Dining and Restaurant Chains	Casual Dining and Restaurant Chains	Casual Dining	Fine Dining
**Totalscope 3 emissions in t CO_2_eq.**	31,255,912 (99 %)	1,073,761 (99 %)	681,192 (99 %)	188,105 (85 %)	67,557 (85 %)	29,971 (95 %)	25,394.15 (96 %)
**Supply chain emissions (food and beverage)**	22816816 (73 %)	671101 (62.5 %) From food and beverage purchases	231605 (34 %) Supply chain emissions	Largest supply chain emissions - food, drink, transport, and distribution.	41885 (62 %)	19481 (65 %)	Not broken down
**Supply chain emissions (non-food & beverage**	Not broken down	165359 (15.4 %) Non-food and beverage purchases	Not broken down	Not broken down	6080 (9 %)	Not broken down	Not broken down
**Energyconsumptionclientsites**	Not broken down	151400 (14.1 %) From energy used in kitchens at the client's sites	361032 (53 %) Client site energy consumption	Not broken down	10133 (15 %) Fuels and electricity	Not broken down	Not broken down
**Travel and commuting emissions**	Not broken down	17180 (1.6 %) From transport and travel	61307 (9 %) Employee commuting & business travel	Not broken down	4053 (6 %) Commuting and business travel	Not broken down	Not broken down
**Other emissions**	Not broken down	68721 (6.4 %)	20435 (3 %)	Not broken down	5405 (8 %)	Not broken down	Not broken down

a 17 additional fine dining restaurants were selected during the grey literature search; however, none of these currently disclose this information. A list of the names of 17 fine dining restaurants can be found in Appendix H.

### 3.2 GHGE data

**Fig. 1:**
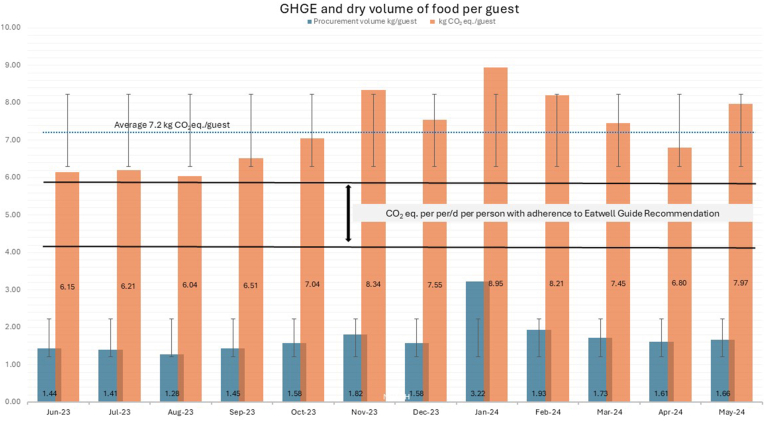
Volume-based GHGE per guest (kg CO_2_ eq./guest) and food procurement volume per guest (kg/guest) for each month. The blue line is the annual GHGE average (7.2 kg CO_2_ eq./guest). Black lines represent the estimated range of daily GHGE per person with adherence to the Eatwell guide (British Dietetic Association, 2020); low range 4.1 and high range 5.8 kg CO_2_ eq. per per/d, respectively.

Monthly and annual GHGE associated with food procurement were estimated based on the volume of food procured (kg/guest). Monthly variations for volume of food procured and associated GHGE were observed (Fig. 1). T-tests for GHGE variation between months showed no significant differences. The average GHGE per guest (7.27 kg CO_2_ eq./guest) exceeded the GHGE range of those for the Eatwell guide (4.1–5.8 kg CO_2_ eq./person/d) (British Dietetic Association, 2020) every month. The monthly average volume of food procured per guest was 1.72 kg/guest.

The restaurant changes its menu quarterly. The average GHGE per menu season was calculated, as shown in Appendix D. The summer months had the lowest average GHGE.

The top ten GHGE categories (of 40) and GHGE/month associated are displayed in Table 2. GHGE hotspots of bovine meat (1.74 kg CO_2_ eq./guest), lamb and mutton (0.92 kg CO_2_ eq./guest), poultry (0.50 kg CO_2_ eq./guest), crustaceans (0.45 kg CO_2_ eq./guest) and fish (0.38 kg CO_2_ eq./guest) totalled ∼71 % of all food-related embedded GHGE. The annual GHGE contribution of all 40 GHGE categories is found in Appendix F.

Table 2 shows that the purchasing volumes for the ten highest GHGE-contributing food categories are lower than their GHGE contribution, except dairy milk. The complete list of food categories and their proportionate GHGE versus total purchasing volume can be found in Appendix F.

**Table 2:** Top ten GHGE category percentage contributing to annual emissions and their volume percentage contribution.

GHGE food categories	GHGE percentage contribution	Volume percentage contribution
Bovine meat	30.8 %	5.0 %
Lamb and mutton	16.4 %	1.5 %
Poultry meat	8.9 %	6.0 %
Crustaceans	8.0 %	1.9 %
Fish	6.8 %	4.4 %
Game	5.8 %	0.8 %
Cheese	4.0 %	3.2 %
Pig meat	3.3 %	1.4 %
Dairy milk	2.6 %	6.0 %
Chocolate	2.4 %	0.5 %
**Other**	**11 %**	**69.3 %**
**Sum**	**100 %**	**100 %**

**Table 3:** GHGE contribution for different food groups to annual GHGE for 4000 rounds of Monte Carlo calculations.

*Procurement category*	*GHGE Meat*	*GHGE Fish*	*GHGE Dry*	*GHGE Dairy*	*GHGE Fruit*	*GHGE Other*	*GHGE Bread*	*GHGE Beer*	*GHGE* *Soft Drinks*	*Total GHGE*
**Minimum [t CO_2_ eq.]**	482.85	94.57	76.88	41.91	25.05	1.36	3.30	0	0	878.24
**Maximum [t CO_2_ eq.]**	1037.07	245.36	218.55	96.26	50.16	30.08	9.23	0.11	0.01	1512.33
**Standarddeviation [t CO_2_ eq.]**	74.71	21.46	22.50	7.44	3.58	4.17	0.87	0.02	0	81.84
**GHGE values without Monte Carlo [t CO_2_ eq.]**	550.60	120.92	76.44	51.68	28.14	8.88	4.26	0.04	0	1125.89

### 3.3 Monte Carlo sensitivity analysis results

**Fig. 2:**
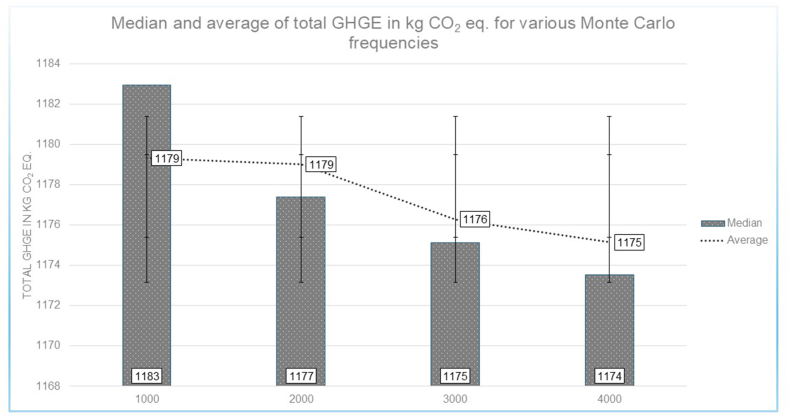
Monte Carlo simulation summary of the annual GHGE with increased frequency of randomisation. The bars represent median annual GHGE t CO_2_ eq. for 1000, 2000, 3000, 4000 simulations, the line is the average yearly GHGE t CO_2_ eq., and the error bars are the standard deviation.

The median, average and standard deviations of total annual GHGE in kg CO_2_ eq. for 1000, 2000, 3000 and 4000 Monte Carlo simulations are shown in Fig. 2. The total annual GHGE after 4000 repeated Monte Carlo simulations was 1175 t CO_2_ eq.; this was higher than 1130 t CO_2_ eq. per year of the initial assessment.

**Fig. 3:**
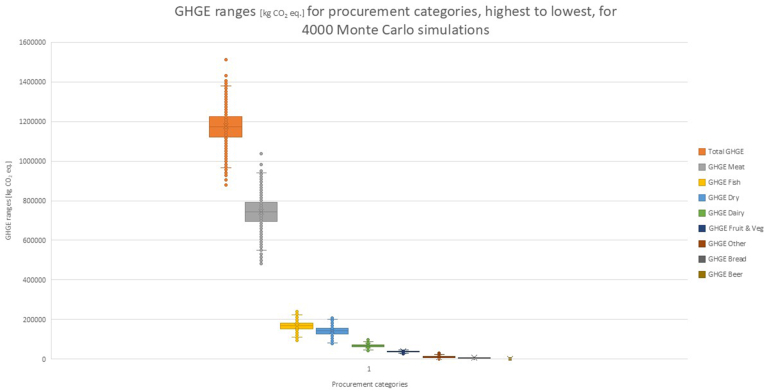
Range of GHGE contribution for different food groups to annual GHGE for 4000 rounds of Monte Carlo calculations. The annual GHGE range was [878.24–1512.33 t CO_2_ eq.]. The box plot graph shows outliers for each of the food groups as well as the standard deviation.

The range of GHGE contributions for seven food categories for 4000 Monte Carlo simulations is shown in Fig. 3. The largest variation of GHGE contribution, or the greatest unknown, is in the meat category, followed by fish. The range for beer and soft drinks has been omitted from the graph because the overall contribution was minimal.

**Fig. 4:**
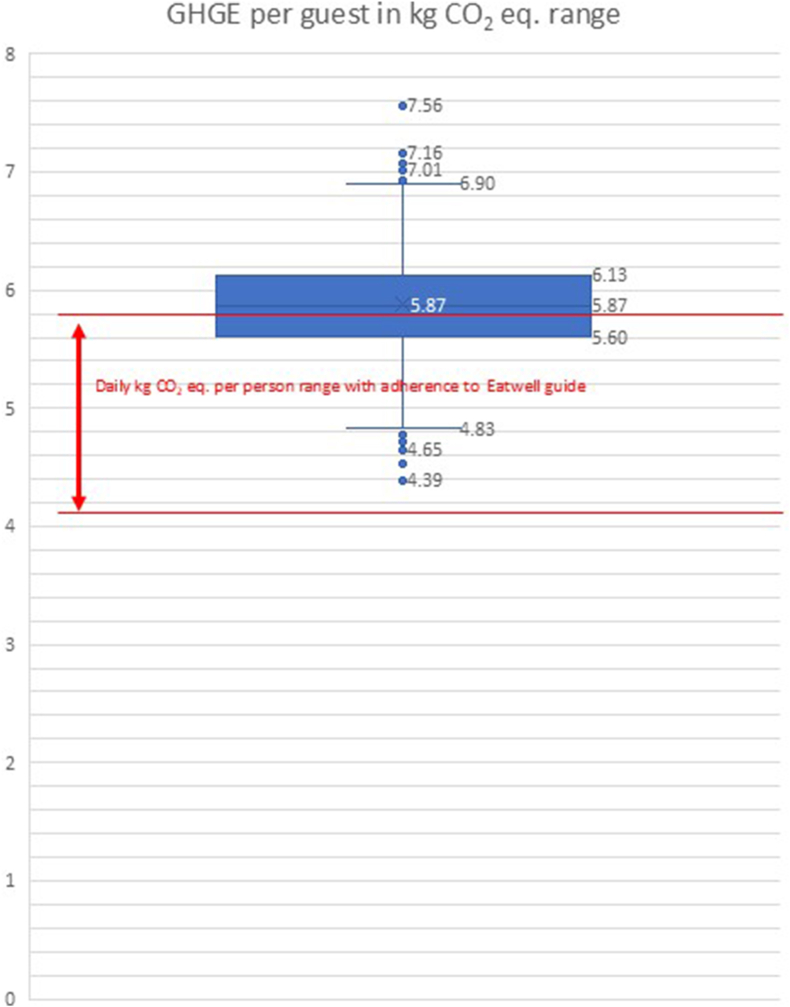
Box Plot for the GHGE kg CO_2_ eq./guest for 4000 rounds of Monte Carlo calculations. Red lines represent the estimated range of daily GHGE per person with adherence to the Eatwell guide (British Dietetic Association, 2020); it ranges from 4.1 to 5.8 kg CO_2_ eq./person/d. Outliers are shown, and the blue line is the STD.

The average GHGE per guest in kg CO_2_ eq. and standard deviation after 4000 rounds of Monte Carlo simulations is visualised in Fig. 4. The GHGE range associated with adherence to the Eatwell guideline (British Dietetic Association, 2020) is mapped into this figure, indicating that the lower end of the standard deviation would fall within this range. The analysis showed that GHGE per guest consistently exceeded the Eatwell guide range of 4.1–5.8 kg CO_2_ eq. per person per day (British Dietetic Association, 2020), even under conservative estimates. Outliers and standard deviation indicated moderate variability across 4000 simulations, but the overall trend remained above recommended sustainable diet benchmarks. Additional details and the box plot illustrating these distributions are provided in Appendix F, Fig. 4.

Table 4 compares the food categories and recommended percentage diet contribution from the Eatwell guideline (British Dietetic Association, 2020) with the percentage of purchase volumes for these food groups in annual restaurant purchases, showing that the food group mix varies from the Eatwell guide recommendations.

**Table 4:** Proportion of Eatwell guide categories for annual food procurement.

Eatwell guide categories	GHGE categories included	% GHGE category volume contribution (% GHGE contribution)	Eatwell guide recommendation contribution
**Starchy foods**	Wheat and rye products, rice, maize and maize products, root vegetables	**21 %** (2.1 %)	**38 %**
**Fruit and vegetables**	Other vegetables, tomatoes, other fruit, citrus fruit, other pulses, brassicas, berries and grapes, onions and leeks, apples, bananas	**32 %** (3.5 %)	**40 %**
**Dairy and alternatives**	Cheese, dairy milk, plant milks	**10 %** (6.6 %)	**8 %**
**Protein**	Bovine meat, lamb and mutton, poultry meat, crustaceans, fish, game, pig meat, egg and egg products, nuts, tofu and soy products	**23 %** (81.2 %)	**12 %**
**Oils and spreads**	Olive oil and olives, rapeseed oil, Sunflower oil	**8 %** (3.3 %)	**1 %**
**Other**	Chocolate preserves, cane sugar, coffee, wine, spices and seasonings, Tea, beet sugar, carbonated drinks and soft drinks, food additives	**6 %** (3.2 %)	**1 %**

### 3.4 Food waste assessment results

**Fig. 5:**
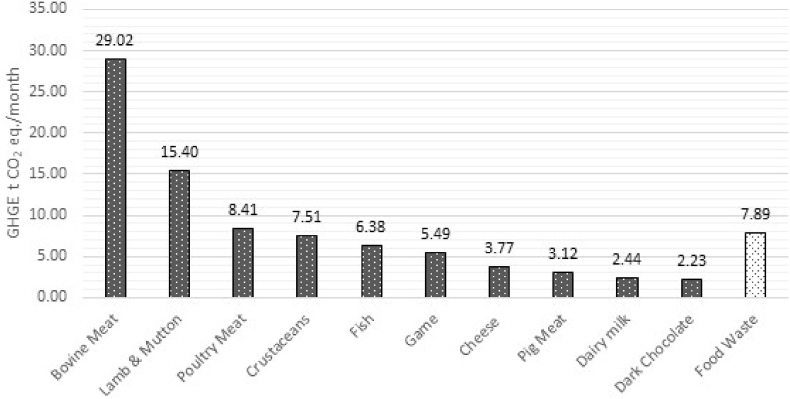
GHGE category emissions contribution compared with the GHGE of food waste Sep 23 – May 24. Food waste estimation is based on a different assessment method. See Appendix B and C for reference.

It was calculated that 1.71 % of the food procured over the 9 months (Sep 23-May 24) was wasted. Only nine months were calculated because there was no available procurement data beyond May 24 at the point of assessment. The restaurant purchases large quantities of animal bones to produce stocks and sauces. These are not recorded as food waste; however, if the purchased volumes were included in the food waste analysis, the food waste proportion would increase to 3.06 %. The associated GHGE of food waste for Sep 23–May 24 would be the fourth most significant contributor to GHGE, as shown in Fig. 5.

Using the average volume of food procured per guest of 1.72 kg (see *3.2. GHGE data*), deducting 3.6 % food waste and a further 25 % for cooking losses this equals around 1.25 kg of food was served per guest.

Appendix D displays the associated GHGE of food waste alongside the seasonal GHGE per guest for the periods for which food waste and procurement data were aligned at the time of data collection. The food waste emissions add to the average GHGE per guest of the food consumed.

## 4 Discussion

### 4.1 Study key findings

This study provides the first volume-based assessment of food procurement GHGE in a UK fine dining restaurant and examines food waste emissions over 52 weeks. Food waste and GHGE comparisons were limited to nine months due to misaligned data collection periods, and the scope excluded beverages, sundries, and transport emissions.

This study found that bovine meat, lamb, poultry, crustaceans, and fish accounted for 71 % of food-related GHGE, with an average of 7.27 kg CO_2_ eq. per guest, exceeding the GHGE associated with a diet following the Eatwell Guide targets for every month of the year. Food waste contributed as the fourth-largest GHGE source. A Monte Carlo analysis highlighted the sensitivity of GHGE estimates to emissions-factor variability, revealing a potential underestimation of around 4 % and confirming that meat categories contribute the greatest uncertainty. The hypothesis that monthly GHGE would vary was supported, but differences were not statistically significant.

### 4.2 Interpretation of findings and literature comparison

GHGE hotspots in this study are similar to those of other caterers (Mistretta et al., 2019), with bovine meat, lamb and mutton, poultry, crustaceans and fish contributing to over 71 % of the total annual GHGE of the food purchases. Mistretta et al. (2019) estimated the GHGE contribution of different foods in school catering in Italy, with meat and fish contributing 58.1 %, this could be due to cultural differences in overall animal protein consumption, however animal protein and energy intake in Italy have been reported to be higher than in the UK at population level (Ritchie et al., 2023). The procurement data in this study includes large amounts of inedible animal products such as beef bones, fish bones and langoustine shells, which are used for stock making at the restaurant, this might partially explain the higher proportion of animal products procured. Further animal protein might be preferred in a fine dining setting and thus contributes much more to GHGE. The higher associated GHGE from animal products also appears to support the findings of Biermann and Rau (2020), which suggest that people are more likely to choose meat when eating out.

Jungbluth et al. (2016) estimated the average canteen meal to be 4.1 kg CO_2_eq.; this is below the calculated average GHGE per guest throughout the year in this restaurant. Additionally, the lower range of the annual GHGE per guest (5.6 kg CO_2_ eq.) estimated in the Monte Carlo simulation is above the value. The procurement data contained inedible animal products, and it is not clear whether such products were included in Jungbluth et al.’s (2016) assessment. Jungbluth et al. (2016) also warn that the contribution of alcoholic beverages could increase the overall GHGE of the evaluation, but this study excluded table-served drinks, so the proportion of wine and spirits in the data set was below 1 % of the total volume. The methodological approach could contribute to the difference, Jungbluth et al. (2016) included coffee, other materials such as serviettes and food disposed in their life cycle assessment. assessment. Lastly the proportion of meat in their study appears to be below the average serving size of meat (180g per main meal) in this restaurant. It is therefore possible that both the methodological approach or dining in a fine dining restaurant is associated with higher carbon emissions than eating a canteen meal. These results might be explained by larger portions and/or more carbon-intensive ingredients per person in fine dining restaurants. Guests, for example, often eat several courses in restaurants, increasing the potential for large portions and higher GHGE. A different proportion of foods with high GHGE factors are eaten in a fine dining context, such as bovine meat, lamb and mutton being consumed more (Biermann and Rau, 2020), would also increase GHGE per guest.

Comparing these studies, it is apparent that the functional unit ‘a meal’ differs between studies (Cerutti et al., 2018; Jungbluth et al., 2016; Mistretta et al., 2019; Wickramasinghe et al., 2016). Articles from the academia and non-profit catering sector tend to report GHGE per meal and kg of food. We assume this preference is linked to the different business models, and it is something academics may need to explore and discuss in future to minimise these methodological and science communications tensions. The portion size and composition of a meal can vary between canteen food offers and restaurant meals (Roberts et al., 2018), thus we offered 4.79 kg CO_2_ eq. per kg of food procured (±0.54) as a unit which allows cross comparison between different types of catering businesses. However, from a restaurant's perspective, the GHGE per guest are perceived as more meaningful than per kg of food purchased because the restaurant management reviews other KPIs based on guest metrics, such as average spend per guest, average food cost per guest, average food waste per guest, etc.

Our hypothesis that the GHGE associated with food procurement varies seasonally was confirmed; however, the monthly variation was not statistically significant. Further studies might be able to explore this and add to research on dietary patterns in temperate climates, such as those from Spence (2021) and Folwarczny et al. (2022).

The summary of other scope 3 emissions data from UK HaFS businesses shows that food and beverage procurement are major contributors to GHGE. For example, Compass UK & Ireland (2024) report 62.5 % or Hawksmoor (2024) up to 96 % of their scope 3 emissions from food and beverage purchases. This grey literature review also confirmed that there is scarce evidence for food procurement GHGE assessments in UK catering SMEs. The GHGE assessment has been documented step by step, including decisions made on boundaries, purpose and calculation methods. There is uncertainty in matching emissions factors for some foods. For example, precise farming practices of some foods were unknown or composite foods such as ready-made sauces may lack specific emissions factors. Studies show that GHGE for beef vary depending on the farming inputs and grazing methods (Cusack et al., 2021), which are not always detailed in the procurement system. Therefore, a cautionary approach was employed, applying the highest GHGE factors for those commodities. Records of the selected emissions factors were kept for future analysis. In addition, a Monte Carlo simulation was carried out to understand the possible data uncertainty. The Monte Carlo simulation suggests an underestimation of the annual GHGE calculation and confirms that the greatest ‘insensitivity’ of emissions factors is found in the meat category of this study. The variation between the GHGE per annum of the assessment and the annual GHGE after 4000 repeated Monte Carlo simulations was 45.58 t CO_2_ eq. (4 %), with more robust emissions data, this limitation could be overcome in time. However, given that five GHGE categories make up 71 % of the yearly GHGE contribution (Table 2), working to reduce the volume of those categories procured could decrease the overall GHGE contribution despite the limitations of the current emissions factor data.

### 4.3 Implications and opportunities

The British Dietetic Association (2020) estimates the recommended healthy Eatwell guideline diet has a 4.1–5.8 kg CO_2_ eq./person/d. The Monte Carlo sensitivity analysis ascertains that the GHGE per guest at the fine dining restaurant currently generally exceeds the estimated range of daily GHGE per person by Eatwell Guide diets (British Dietetic Association, 2020). However, the GHGE per guest estimated here are for one daily dining occasion rather than the total daily dietary intake. The inedible animal products in the procurement data (such as beef, fish bones and langoustine shells) might explain some of this difference. Further, a simple dietary gap analysis was carried out to compare the proportions of different food groups in the procurement data with the recommendations of the Eatwell guide. As Table 4 shows, currently, the proportion of Eatwell guide categories purchased by the restaurant differs from the recommended proportion contribution. For example, the protein proportion in the procurement data was 23 % but the recommended protein contribution in the Eatwell guide is 12 %. This category contains the bovine meat, lamb, poultry, crustaceans and fish, the five largest GHGE contributors as shown in Table 2. Thus, there is an opportunity to revise the restaurant's food offer and food procurement to align it more closely with the Eatwell guideline food composition, and overall GHGE emissions could be reduced. Food offer or menu redesign has been cited elsewhere as a suitable strategy to reduce GHGE associated with dining out (Pollicino et al., 2024; Stiles et al., 2022). A further driver of the higher GHGE associated per guest could be the size of the meal. The calculation for the volume of food per guest estimated 1.25 kg of food per guest. WRAP (2020), for example, recommend using 420 g as the average meal size weight for assessments of out-of-home settings. In reference to this, it would imply that all guests have around three average meals at a single dining occasion, highlighting that there is a potential for very large portion sizes served to guests, also reported in nutritional studies (Muc et al., 2019). However, there are a few data limitations to consider. While the reported food waste adjusted the weight, for cooking losses, the data for the guest numbers is not drawn from the same database, and while there are efforts to record every meal served, staff food, for example, is not recorded through this system. After a conversation with the executive chef, it was confirmed that around 500 additional meals are being made weekly from the food procured. The volume of food per ‘guest’ and GHGE per guest were recalculated by adding 2000 ‘guests’ per month. This information is provided in Appendix G. The volume of food per guest dropped to 1.1 kg after food waste and cooking losses had been accounted for; the average GHGE per guest was found to be 6.27 kg CO_2_ eq. in this adjusted calculation.

### 4.4 Food waste data relevance and sensitivity

The food waste percentage of 1.71 % over 36 weeks is significantly below those reported elsewhere, such as the 18 % in HaFS stated in the latest UK review by Malik et al. (2024). The business has actively set food waste reduction targets since 2018. Food waste may be below the industry average, possibly due to the restaurant's active reduction targets and possible underreporting. It is notable that despite these efforts, the GHGE per month for food waste were still the fourth largest contributor to GHGE. Animal bones and frying oil were not recorded as these food waste streams were deemed unavoidable by the food business (Nicholes et al., 2019). Liquid food waste was drained into wastewater, such as milk or juice, and occasionally, employees failed to keep accurate records. Underreported food waste results in lower GHGE calculated for food waste. However, this marks the first 52-week consecutive food waste record for a fine dining restaurant in the United Kingdom, using the Guardians of Grub Food Tracker (WRAP, 2017). It confirms the tool's usability for daily recording but also highlights its limitations.

### 4.5 Feasibility of WRAP methodology for routine assessments

Detailed process records would enable the business to repeat the assessment annually, set reduction targets, and review progress using process mapping. Process mapping can improve efficiency, although savings are difficult to predict. Realistically, a restaurant would prefer to upgrade its procurement system to carry out recipe-based GHGE assessment alongside the calculation of food cost and the nutritional content of dishes, which is already possible with some systems. This might mean that some of the decision-making processes, such as unit assignments and emissions factor allocation, could be handled by specialists working for the procurement company. At the same time, the calculation of GHGEs could be done in conjunction with exploring other business metrics, such as profit margins.

Summarising food waste data from multiple trackers further increased the risk of errors. If a proposed monthly or annual tool was embedded in the procurement system as a record, some of these data entry risks could be mitigated, and a linkage to food purchases could be established. Alerts for missing or unusual records and regular training on food waste recording could improve performance tracking.

### 4.6 Study limitations

This study faced several limitations that may influence the interpretation of results. First, the accuracy of the GHGE was constrained by the quality of data in the procurement system and the ability to link emissions factors to the food procured accurately. Several data points were excluded because we found entries that were too large to have been purchased, as well as lines for which we could not assign emissions factors (e.g., Invoice adjustment or food additives, such as xanthan gum, for which the reference database lacked emissions factors). This contributes to the underestimation of the overall GHGE of the food procured. However, the study enables the business to understand which foods have the most significant impact on GHGE. The business can now focus on those GHGE hotspots in its efforts to redesign its menus and work with its procurement team to buy ingredients produced in ways that have a lower GHGE impact.

Second, food waste data collection was delayed until September 2023 due to staffing and operational challenges, resulting in only nine months of aligned procurement and food waste data. This limited direct comparisons between procurement and waste-related emissions. Furthermore, the Guardians of Grub tool records data in four-week periods, whereas procurement reporting is monthly, complicating temporal alignment.

Third, manual food waste recording and subsequent transfer of paper records into Excel introduced risks of data entry errors and inconsistencies, particularly when multiple staff were involved. A fully integrated digital system linking procurement and waste data could reduce these risks and improve accuracy.

Finally, this assessment represents a partial scope 3 analysis, excluding beverages, sundries, and transport emissions. While these omissions may underestimate total emissions, the study still identifies major GHGE hotspots and provides actionable insights for menu redesign and procurement strategies.

## 5 Conclusion

We utilised a restaurant's annual food procurement data and the WRAP Emission Factor Database v2.0 to conduct a volume-based GHGE assessment for all food purchases made through system. A Monte Carlo sensitivity analysis revealed potential underestimation of around 4 % for annual GHGE, with meat GHGE contributing to the greatest variability in the data. The analysis revealed seasonal variations in GHGE of food procurement, potentially related to seasonal menu changes and consumer preferences regarding dietary patterns and portion sizes. This could be investigated using relevant sales data and may present opportunities for intervention. Despite the risk of underestimation of annual GHGE the assessment enables the business to track emissions reduction goals and to design evidence-based, targeted interventions. However, this means the assessment needs to be operationalised and done regularly.

The established business practice of recording food waste in this restaurant enabled the incorporation of this data into the analysis. However, an introduction of a monthly tracker for food waste, featuring a similar feature to the Guardians of Grub tool, would provide better data comparability as well as using regular opportunities to brief those collecting the data on accurate data capture.

This case study supports collaboration with primary producers for improved data accuracy and emissions measurement, specifically for meat and fish products. While every effort was made to select the most appropriate emissions factor, there was uncertainty about farming practices, such as whether the chicken and pigs were soy-fed, which can influence the emissions factor.

Nevertheless, we believe the hotspots identified in this assessment present an opportunity for a GHGE baseline, which will inspire targeted GHGE reduction and seasonal opportunities for menu engineering, specifically when compared with the recommended percentage food group contributions of the Eatwell guide.

This dataset enables future work, such as ANOVA tests, to assess whether GHGE categories contribute significantly differently to overall GHGE. Another opportunity is grouping the month into seasonal periods, such as the menu change, to review the impact of seasonality on food procurement and associated GHGE. Removing inedible animal proteins from the data set could also make the data more comparable with the Eatwell guideline and other studies, such as the one from Jungbluth et al. (2016). Repeating the analysis with the procurement data of the following year will support insights into procurement changes and the potential to impact the GHGE hotspots. Furthermore, there is potential to calculate the business's specific emissions factor from its annual GHGE data for food waste.

## CRediT authorship contribution statement

**Andrea Zick:** Writing – original draft, Visualization, Validation, Project administration, Methodology, Investigation, Formal analysis, Data curation, Conceptualization. **Ximena Schmidt R:** Writing – review & editing, Validation, Supervision, Resources, Data curation. **Christian Reynolds:** Writing – review & editing, Visualization, Validation, Supervision, Resources.

## Declaration of generative Ai and Ai-assisted technologies in the Writing process

During the preparation of this work, the author(s) used Grammarly Pro in order to improve the grammar and spelling of this paper. After using this tool/service, the author(s) reviewed and edited the content as needed and took full responsibility for the content of the published article.

## Funding declaration

This research was funded by the UK Food Systems Centre for Doctoral Training (Project Reference: V011391/1).

## Declaration of competing interest

The authors declare the following potential competing interests.

• Andrea Zick: consultancy for Chefs Forum & NHS England; stock ownership in The Ferm; ambassador roles with Be Inclusive Hospitality and WRAP; advisory roles with Feast with Us and Lewisham Good Food Steering Group; PhD is funded by the UK Food Systems Centre for Doctoral Training (The Partnership for Sustainable Food Future Centre for Doctoral Training (PSFF-CDT); Project Reference:BB/V011391/1.
• Dr Ximena Schmidt R.: Advisory/director roles at Emissions Insight Ltd (UK) and ClearPrint SpA (Chile); consulting for WRAP and other sectors; speaker at industry events.
• Dr Christian Reynolds: Advisory roles with Nutrition Society, IFST, and ISO and BSI committees; consulting for WRAP, Zero Waste Scotland, DEFRA, and FSA; pro bono expert advisory and speaking engagements; research funding from Alpro Foundation (€49,858), the Healthy Soil, Healthy Food, Healthy People (H3) project (Project Reference: BB/V004719/1). These are funded by the ‘Transforming UK Food System for Healthy People and a Healthy Environment SPF Programme’ delivered by UKRI, in partnership with the Global Food Security Programme, BBSRC, ESRC, MRC, NERC, Defra, DHSC, PHE, Innovate UK and FSACR is funded by UKRI and NIHR as part of the Building A Green Future strategy the THRIVING Food Futures research hub(MR/Z506485/1) Finally Dr Christian Reynolds receives funding by UKRI (through the Building a green future and Building a secure and Resilient world cross UKRI themes), Defra and NERC and administered by NERC on behalf of the partners by the Joined up Landscapes (Project Reference: APP43555 UKRI1280).

## Figures and Tables

**Table 5:** Screening inventory for Scope 3 assessment

Research objectives	Business pbjectives	Why only s*cope 3 Category 1* purchased goods and services analysis?	Which boundary?	Which functional unit?
Create a food emissions data set for a fine-dining restaurant.	Maintain a 50 % food waste reduction against the baseline	This is likely the most material source of emissions for food and drink businesses.	Procurement data of kitchen purchases only (excludes most beverage purchases of the business, dry catering goods such as clingfilm blue paper or cleaning products)	Cradle to hospitality gate weight-based as opposed to sales-based assessment.
Explore how the GHGE of this business compare to similar contract catering data.	Create a baseline for GHGE of the food procurement to enable goal-setting.	This category is also seen as an area of emissions with the most significant potential for interventions to lower GHGE.	GHGE factors shall ideally be for cradle to hospitality gate, which, according to the WRAP guidance, contains food preparation.	
Explore seasonal differences of GHGE associated with food purchases.	Create a repeatable method to assess progress on GHGE reductions in food procurement.	Retail and hospitality at the end of the supply chain must collaborate with primary producers to reduce scope 3 emissions.		
Understand the most considerable GHGE contributions and how they link to the menu and food offer.	To understand GHGE hotspots.	Increased pressure from customers, investors, and other stakeholders to measure, report and reduce emissions		
Review the food waste data in reference to top GHGE data and the impact improved food waste management may have.				
Review the food waste emissions factors from WRAP for restaurants regarding the procurement data, explore whether these are still fit for purpose given their relative datedness, and propose a specific one for this business.				
Explore how a changed number of guests may have reduced food waste proportionately independently of any practice changes.				

**Table 6:** Units with missing portion size and the portion size decision

Product	Unit	Reference	Comment on decision	Unit weight used for GHGE calculation
Artichoke baby	Each	Başay and Tokuşoğlu (2005)		Each = 94g
Carrots baby	Bunch	Waitrose (2024a)		Each = 150g
Celeriac	Each	Mezeyová et al. (2018)		Each = 650g
Chicken free range whole bird	Packet	Jones et al. (2007)	Chickens were grown for a commercial market to a target ∼ of 2.28 kg	Each = 2.28 kg
Crab soft shell	Each	Fujaya et al. (2020)		Each = 92.5g
Duck confit leg	Each	Albion (2024)	Is a designated supplier of business	Each-280g
Duck egg	Each	Raising Ducks (2024)	FAQ number 17, average calculated.	each = 85g
Egg Ffee range	Each	DEFRA (2020)	Medium egg range used.	each = 57.5g
Blood orange	Each	Legua et al. (2022)	Average weight of 11 cultivars average weights.	each = 158g
Cantaloupe melon	Each	Shafeek et al. (2015)	Average weight is quoted in Table 3.	each = 206g
Coconut fresh	Each	Chan and Elevitch (2006)	Used the lower weight quoted on page 5 because the restaurant receives dried mature coconuts, lighter than the young, fresh green coconuts.	each = 850g
Figs black	Each	Pereira et al. (2015)	Average weight of six cultivars from this article.	each = 75g
Galia melon	Each	Mitchell et al. (2007)	Use the quote for smaller fruits, 0.7–0.9 kg, as it was stated these are preferred for the European market.	each = 800g
Passion fruit	Each	Pathak and Shukla (2006)	Average between 25 and 48g.	each = 36.5g
Pink grapefruit	Each	Sharma et al. (2016)	Average weight of three cultivars calculated.	each = 408g
Quince	Each	Tatari et al. (2024)	Average of reported and cited fruit weights (no 8) was calculated.	each = 250g
Watermelon seedless	Each	Perkins-Veazie et al. (2012)	Average weight between 5 and 7 kg calculated.	each 6 kg
William pear	Each	Colavita et al. (2021)	Average weight between 170 and 350g calculated.	each = 260g
Garlic smoked	Bunch	El-Zohiri and Farag (2014)	Average of mean reported bulb weight ×14 (12–16 bulbs in a bunch)	each bunch = 693.7g
Lamb cannon	Each	AHDB (2002)	UK reference guide so used as in document.	each = 540g
Lotus root	Each	Tian et al. (2009)	Average weight of reported weights Table 1.	each = 469g
Chayote squash -	Each	Qiu and Liu (2022)	Average weight of typical harvest weights quoted.	each = 340.2g
Pigeon whole bird	Each	R and J Yorkshires Finest (2024)	Average weight of this supplier because the pigeons in this restaurant are sourced from Yorkshire.	each = 300g
Pork cutlet french trim	Each	IMS of Smithfields (2024)	Used the average weights of this supplier as they are London-based and have a similar offer to the restaurant.	each = 225g
Quail eggs	Packet of 12	Abadi et al. (2018)	Quality A quail eggs 10–11g each, so average range.	each-10.5g
Salad round lettuce	Each	El-Nakhel et al. (2019)		each- = 130g
Sardines butterfly	Each	Pipers Farm (2024)	Is used because of similar sources as the restaurant.	each = 125g
Scallops hand dived	Each	Fulton Fish Market (2024)	Used because the restaurant sources huge scallops	each = 30g
Squash delicia	Each	Food Bank Central New York (2019)		each = 567g
Venison rump steak	Each	Waitrose (2024b)	Used because of similar sourcing on the website.	each = 250g

**Table 7:** Average seasonal GHGE per guest and GHGE of food waste per guest

Season	Average GHGE per guest [kg CO_2_ eq./guest]	Food waste period	Average GHGE of food waste per guest [kg CO_2_ eq./guest]
Summer (Jul–Sep)	6.32	No data available	No data available
Autumn (Oct–Dec)	7.56	Food waste 2nd Oct - 24th Dec	0.64
Winter (Jan–Mar)	7.96	Food waste 25th Dec - 17th Mar	0.67
Spring (May–Jun)	6.99	Food waste 18th Mar - 9th Jun	0.64

**Fig. 6:**
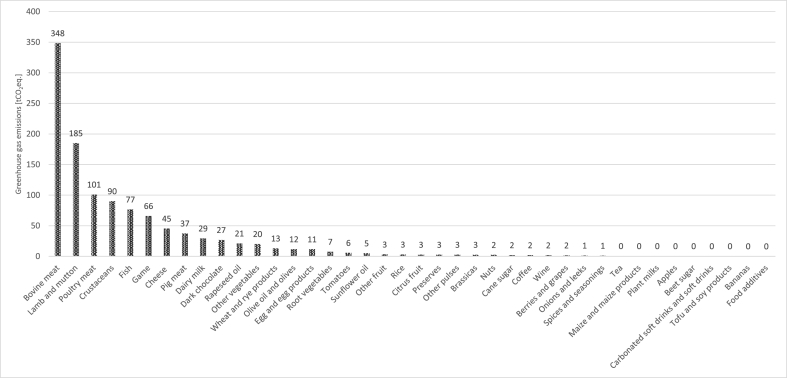
Annual GHGE contribution per food category in t CO_2_ eq.

**Table 8:** Food category GHGE contribution and purchase volume comparison

GHGE food categories	GHGE percentage contribution	Volume percentage contribution
Bovine meat	30.8 %	5.0 %
Lamb and mutton	16.4 %	1.5 %
Poultry meat	8.9 %	6.0 %
Crustaceans	8.0 %	1.9 %
Fish	6.8 %	4.4 %
Game	5.8 %	0.8 %
Cheese	4.0 %	3.2 %
Pig meat	3.3 %	1.4 %
Dairy milk	2.6 %	6.0 %
Chocolate	2.4 %	0.5 %
Rapeseed oil	1.9 %	5.2 %
Other vegetables	1.8 %	14.2 %
Wheat and rye products	1.1 %	7.9 %
Olive oil and olives	1.0 %	2.0 %
Egg and egg products	1.0 %	1.5 %
Root vegetables	0.7 %	12.4 %
Tomatoes	0.5 %	2.3 %
Sunflower oil	0.4 %	1.0 %
Other fruit	0.3 %	3.2 %
Rice	0.3 %	0.6 %
Citrus fruit	0.2 %	3.9 %
Preserves	0.2 %	0.8 %
Other Pulses	0.2 %	1.3 %
Brassicas	0.2 %	2.5 %
Nuts	0.2 %	0.7 %
Cane sugar	0.2 %	1.2 %
Coffee	0.2 %	0.3 %
Wine	0.1 %	1.3 %
Berries and grapes	0.1 %	0.8 %
Onions and leeks	0.1 %	2.4 %
Spices and seasonings	0.1 %	0.2 %
Tea	0.0 %	0.0 %
Maize and maize products	0.0 %	0.3 %
Plant milks	0.0 %	0.9 %
Apples	0.0 %	0.8 %
Beet sugar	0.0 %	0.2 %
Carbonated drinks and soft drinks	0.0 %	1.3 %
Tofu and soy products	0.0 %	0.1 %
Bananas	0.0 %	0.2 %
Food additives	0.0 %	0.0 %

**Fig. 7:**
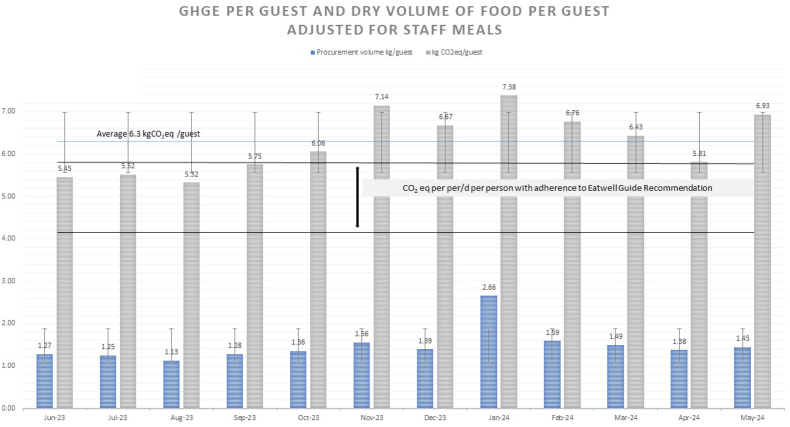
Adjusted volume-based GHGE per guest (kg CO_2_ eq./guest) and food procurement volume per guest (kg/guest) for each month. The blue line is the annual GHGE average (7.2kg CO_2_ eq./guest). Black lines represent the estimated range of daily GHGE per person with adherence to the Eatwell guide (British Dietetic Association, 2020); low range 4.1 and high range 5.8 kg CO_2_ eq. per/d, respectively.

## Data Availability

Data will be made available on request.

## Glossary

GHGE: Greenhouse gas emissions
HaFS: Hospitality and Food Service

## APPENDICES

### APPENDIX B

Procurement GHGE estimation.

I. Download CSV files month by month (Jun 23 – May 24) from the procurement system Tevalis (version tbc).
II. Combine monthly data in one Excel sheet, adding a new monthly column. This led to 6282 data lines for items purchased.
III. Add Excel filters to ease the sorting of data.
IV. Food quantity conversion and data cleaning
  a. For all 'g' units/1000 = 1 kg.
  b. If each item and the unit was specified (e.g. burger 110g each, the formula was built accordingly).
  c. The average weight was used and converted into kg if each unit was specified as a range (e.g. Chateaubriand 750–800g). 8 lines appeared to be affected by human error, possibly having been entered as g or kg when they were, in fact, kg or ea. This was checked with the kitchen team, but it had to be removed due to lack of traceability as it was unclear which unit was correct.
  d. Two thousand twenty lines needed additional investigation for the conversion as they had ambiguous units (such as 'each', 'tin', and 'punnet').
  e. If the unit was ambiguous, a follow-up with the procurement team was done, and the stock system entries were reviewed to determine which ambiguous units had allocated specific weight measurements, which were amended. If various weights were attached to a unit, the average of that unit was used as described above. In these cases, the procurement data did not allow tracing back to a specific supplier and to one size for these units, which would have allowed a more specific analysis.
  f. Liquid units such as beer and soft drinks were transformed from 0.5l to 0.5 kg without viscosity adjustment; however, the formula was adjusted for oil-based liquids with high viscosity. After this step, 30 items were nonspecific in terms of their weight.
  g. To allocate the portion weight of thirty foods not specified by weight in the procurement system comparison were made against academic literature. References were kept in a separate file to allow for retracing the weight allocation.
  h. The outliers (low and high figures) were spot-checked. This highlighted potential human errors (34 lines of data) or items that did not relate to food (e.g., Invoice adjustment 18 lines of data) to ensure they were true or excluded from the calculations/assessments. Both types of data inconsistencies were removed.

The 34 lines contained huge purchase volumes (613940 kg procurement volume in total or 69 % of the total annual procurement volume). For those, the executive chef confirmed these were entry errors (e.g. 2250 kg of Korean pepper paste ordered for a single month), therefore those were excluded.

The executive chef also investigated the 18 lines whose product name was 'Invoice adjustment' and reported that it meant the product ordered was referring to a delivery which was rejected, error some in delivery weight or order volume. These rejected deliveries are not deducted from the order list as individual commodities but processed in bulk at the end of a week. This means it is impossible to retrospectively define the specific product and its weight. The purpose of those lines is to adjust procurement volume and cost of non-delivered items and/or human error data; thus, it is likely that it also refers to human error data (34 lines). Due to the lack of traceable information, these 18 lines were excluded from the calculations.

Finally, 60 lines of data were removed because the order value was zero, which the executive chef confirmed meant these items were not delivered. The exclusion amounts to 2 % of the data or 69 % of the recorded procurement volume, but it can be assumed this would be reduced by the non-volume-specific 18 lines of ‘Invoice-adjustment’.

V. Creation of GHGE look-up and calculation
  a. All named products from the procurement data (raw data) were copied into a new tab, and all duplicates were removed. Each item was matched to a specific emissions factor from the WRAP Emission Factor Database v2.0.
  b. Once these lines were excluded and all items matched with emissions factors, a calculation was applied, multiplying the weight of each item by the appropriate emissions factor.
  c. As there were repeat purchases, 941 unique food and drink commodities were identified and 920 unique emissions factors from the WRAP Emission Factor Database v2.0 (WRAP, 2024) were allocated because for some of the 941 unique food and drink commodities, the same emissions factor was used (e.g. for different types of beans). For 120 lines of data, the allocation of emissions factors was not possible because the database did not include suitable reference factors (Gold leaf envelopes, baking powder, baking powder GF, bicarbonate of soda, citric acid, colour powder black, dried yeast, gelatine powder, honey, ice cube, pectin, salt flake, salt rock, salt table, smoke flavour, xanthan gum, yeast flakes). This increases the data excluded to 4 %. These commodities amount to a procurement volume of 9721 kg (1 % of the total annual procurement volume).
  d. To determine the suitable GHGE per food and drink commodity, these emissions factors were multiplied by the corresponding volumes of these foods.
  e. The monthly and annual totals for each food commodity and the sum of all commodities were calculated.
VI. Analysis of GHGE estimates per month and year.
  a. The monthly total GHGE was adjusted by the monthly number of covers in the restaurant to understand the differences in GHGE contribution per month/per guest.

### APPENDIX C

Food Waste GHGE estimation.

I. Food waste data collection.
  a. Over 52 weeks (Sep 23 – Aug 24), the restaurant's kitchen porters used a designated food waste sheet and weighed and recorded food waste daily.
  b. These records were entered into the Guardians of Grub Food Tracker (WRAP, 2017), which allows the collection of 4 weeks of food waste; thus, the template tracker was duplicated to record 13 periods of 4-week food waste.
  c. These records were combined into one record by copying the total food waste for each 4-week-period into a new Excel document using the spoilage, preparation, plate waste, other food waste and total food waste records for each period.
II. Analysis of food waste data
  a. The number of guests and walk-ins was extracted from the restaurant booking system to ensure seasonal variations of business levels were considered.
III. Food waste GHGE estimation
  a. The waste in tonnes for each four-week period, which was a total of 52 weeks, was multiplied by the food waste emissions factor from the Guardian of Grub tool to calculate the t CO_2_eq. for each four-week-period and a total of 52 weeks.

### APPENDIX F

Annual GHGE contribution per food category.

### APPENDIX H

1. Mildreds,
2. Plates,
3. Daylesford Organic,
4. Land
5. Bubabla
6. Vanderlyle
7. Gauthier
8. Tendril
9. Root
10. Hendersons
11. Silo
12. Apricity
13. St. Barts
14. Petersham Nurseries
15. Wilsons
16. The Evolve Collection
17. JSK Restaurants
